# Supplementary material for: IL-33 enhances Jagged1 mediated NOTCH1 intracellular domain (NICD) deubiquitination and pathological angiogenesis in proliferative retinopathy
Source: Commun Biol. 2022 May 19;5:479. doi: 10.1038/s42003-022-03432-7 (PMC9120174; doi:10.1038/s42003-022-03432-7)

Supplementary Fig. 1

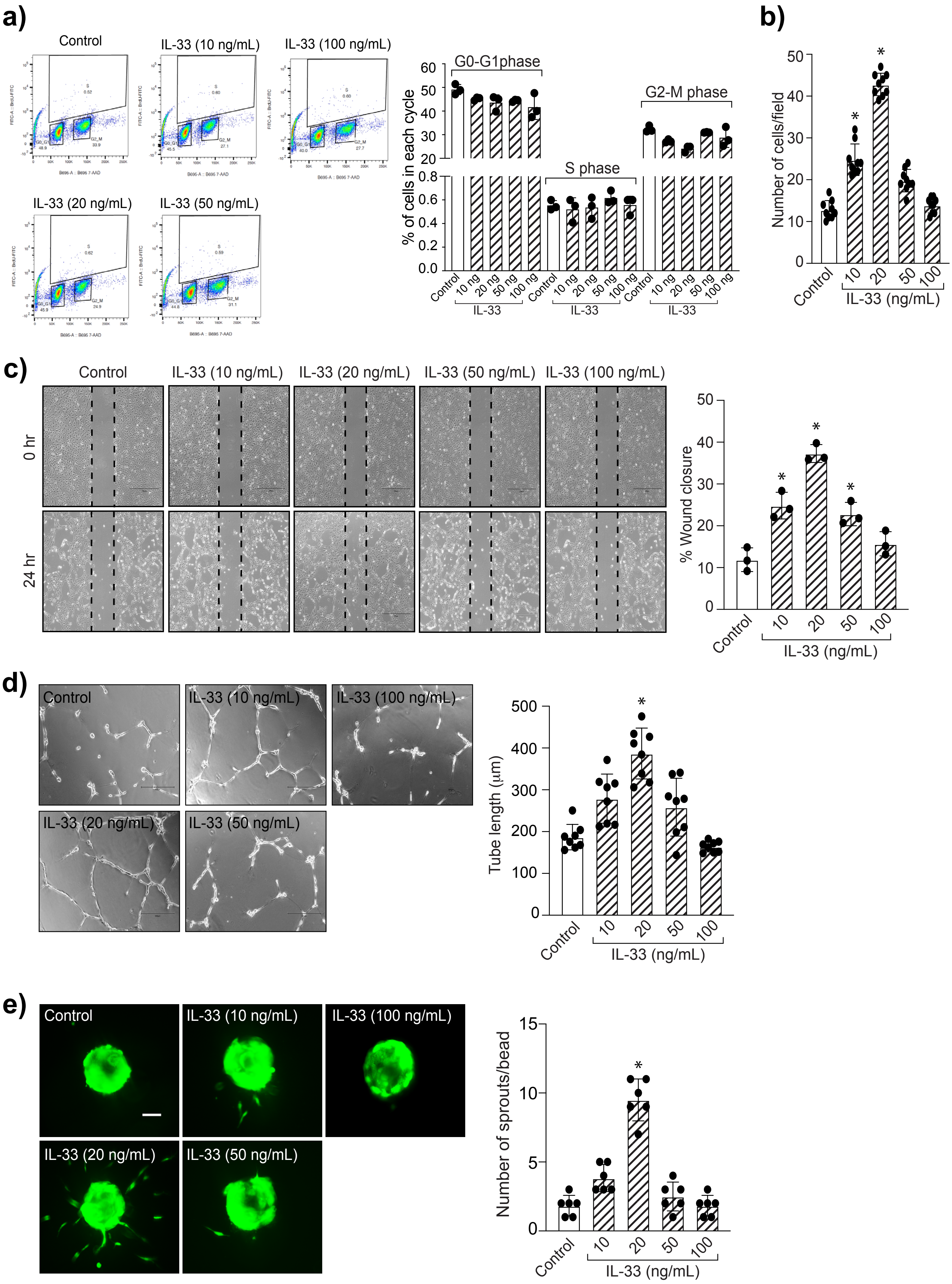

Suppl. Fig. 1: IL-33 induces angiogenic events in HRMVECs at 20ng/mL.

a-e Quiesced HRMVECs were treated with various concentrations of IL-33 and proliferation (**a**), migration (**b** & **c**), sprouting (**d**) and tube formation (**e**) of HRMVECs were measured. The bar graphs show the quantitative analysis of three independent experiments, expressed as Mean ± SD. * $p<0.05$ vs control, one-way ANOVA with Bonferroni's correction. Scale bar represents 50 μm in panel **e**.

Supplementary Fig. 2

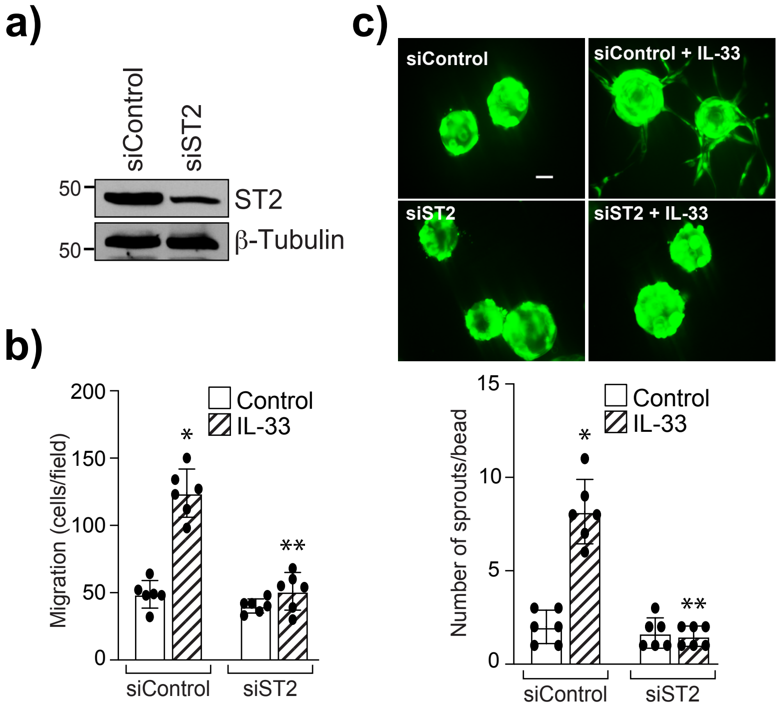

Suppl. Fig. 2: IL-33/ST2 signaling induced angiogenic events in HRMVECs.

a HRMVECs were transfected with control (siControl) or ST2 siRNA (siST2), after 48 hours cell extracts were prepared and analyzed for ST2 levels by Western blotting and reprobed with anti-β-tubulin antibodies. **b-d** After transfection with control and ST2 siRNA, cells were quiesced, treated with or without IL-33 (20 ng/mL) and subjected to migration (**b**), sprouting (**c**) and tube formation (**d**). The bar graphs show quantitative analysis of three experiments, expressed as Mean ± SD. *$p<0.05$ vs control, ** $p < 0.05$ vs control siRNA + IL-33, one-way ANOVA with Bonferroni's correction. Scale bar represents 50 μm in panel **c.**

Supplementary Figure 3: Scans of immunoblots presented in Figure 1, 2, 3 & 5.

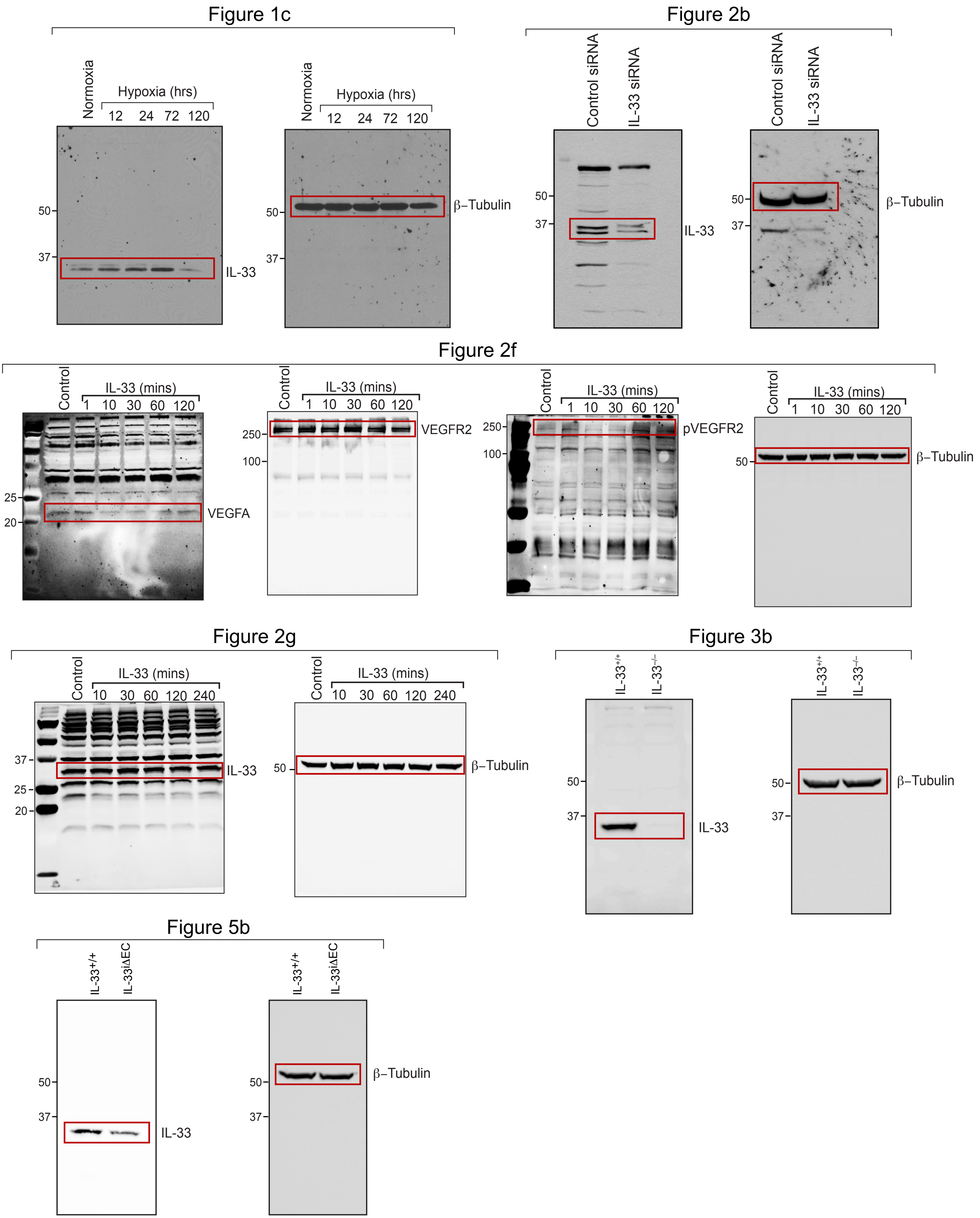

Supplementary Figure 4: Scans of immunoblots presented in Figure 6 a, b & c.

Figure 6a

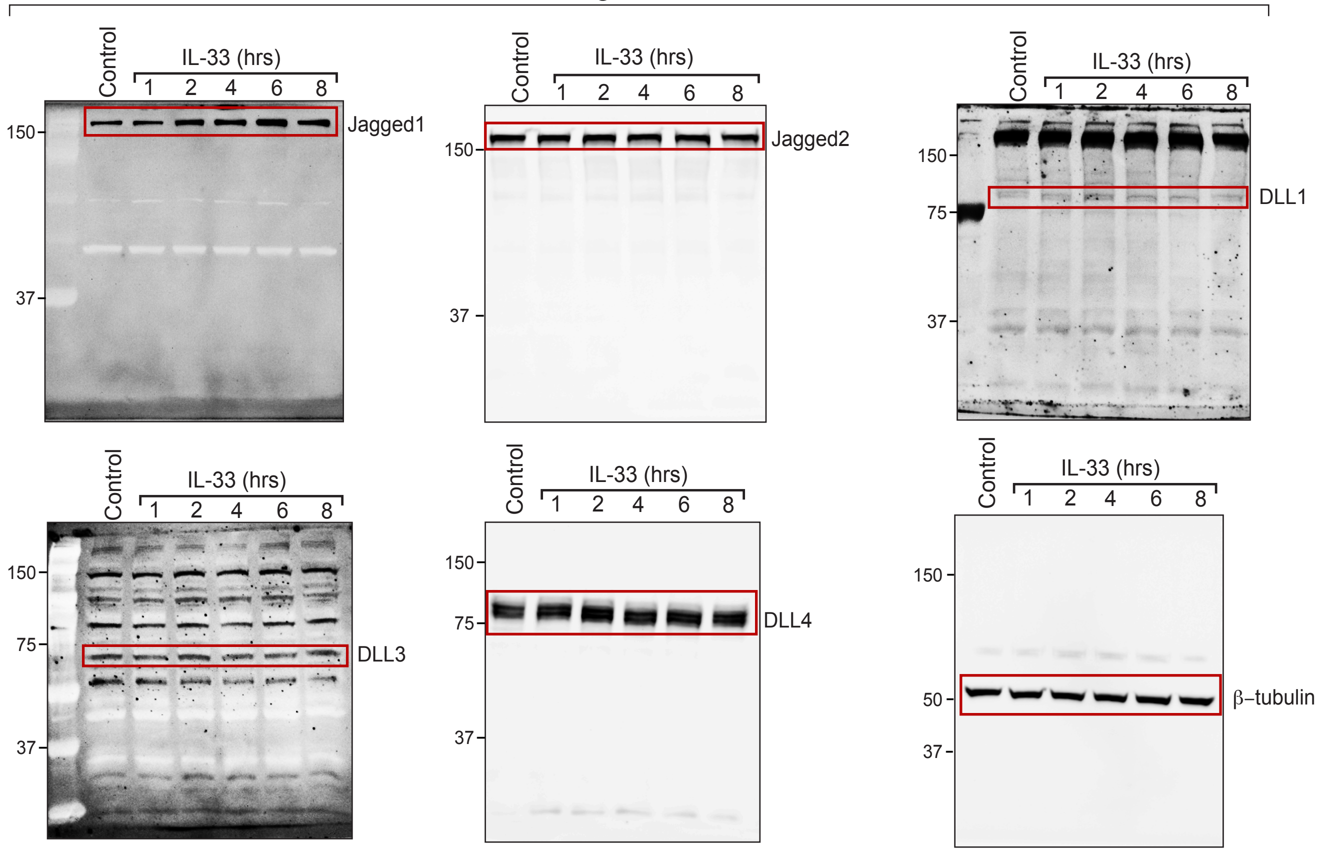

Figure 6b

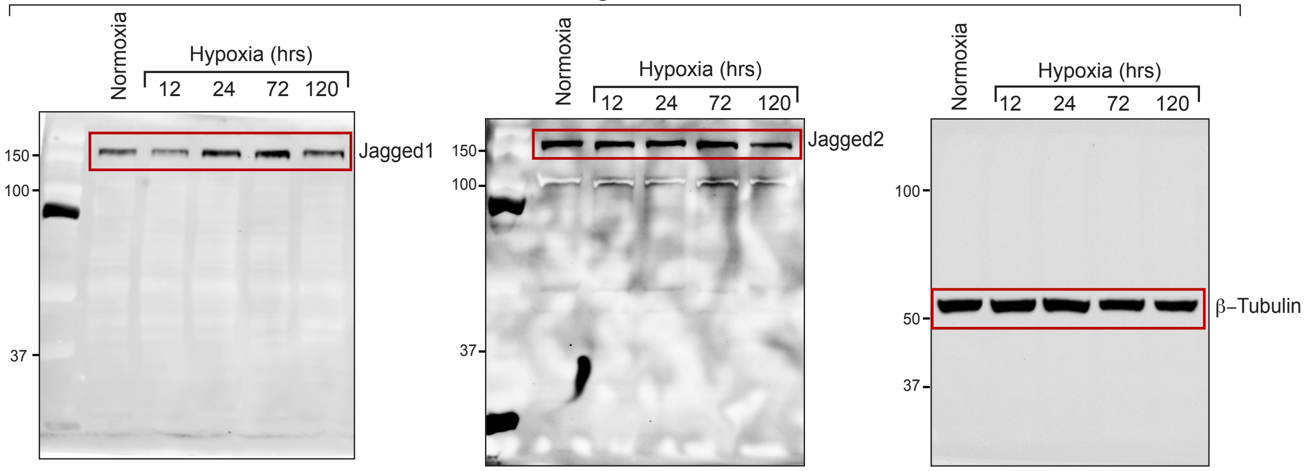

Figure 6c

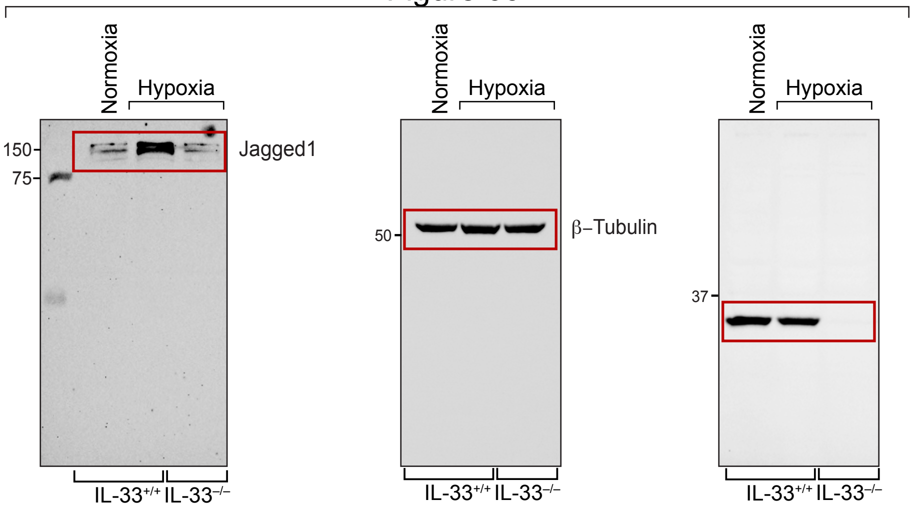

Supplementary Figure 5: Scans of immunoblots presented in Figure 6 d, h & i.

Figure 6d

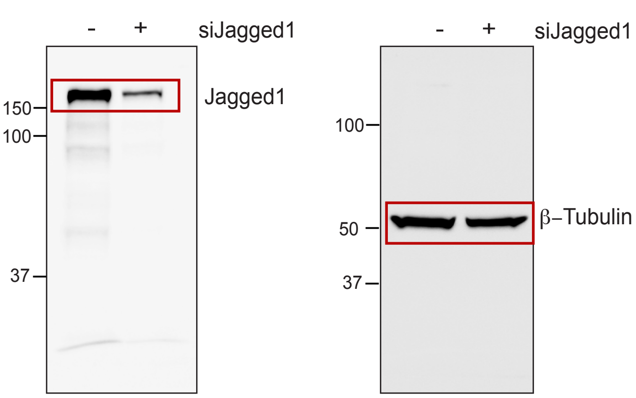

Figure 6h

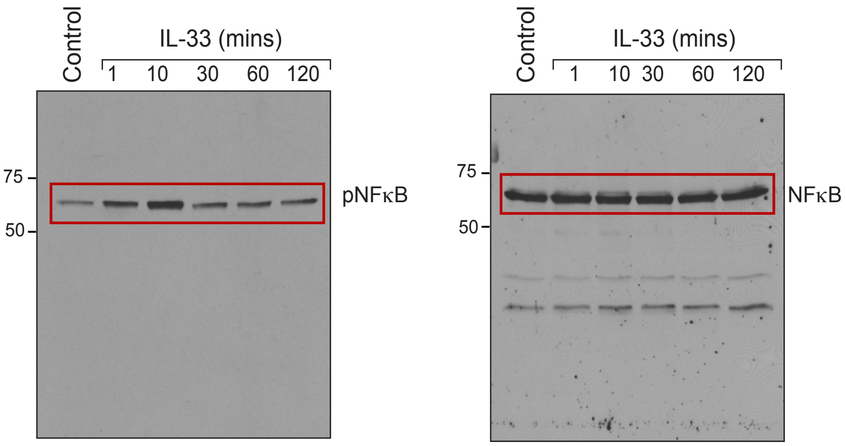

Figure 6h

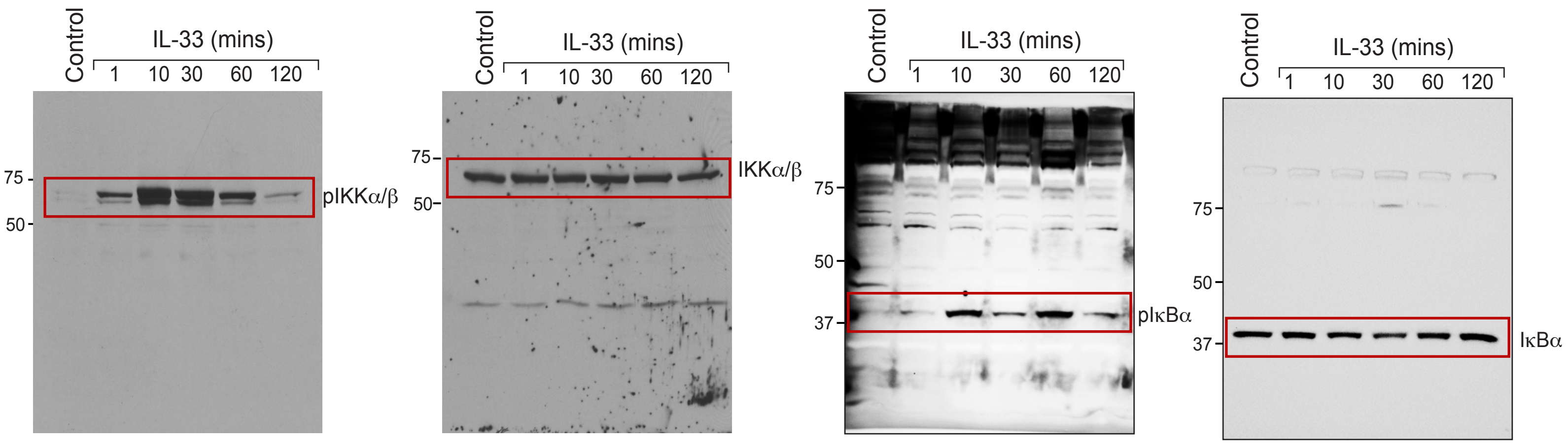

Figure 6i

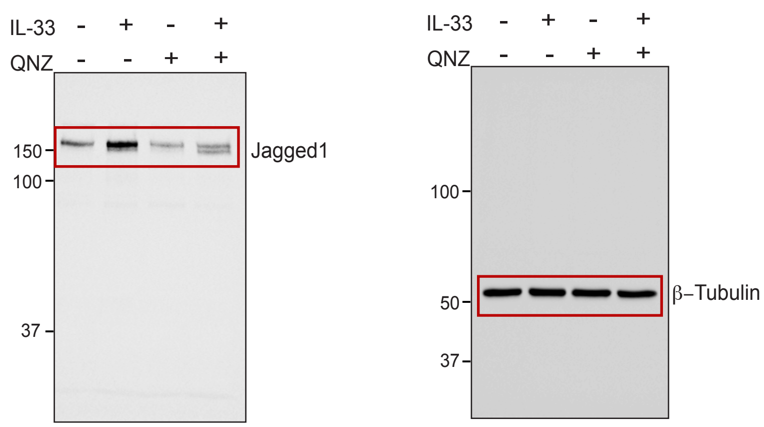

Supplementary Figure 6: Scans of immunoblots presented in Figure 6 j & k.

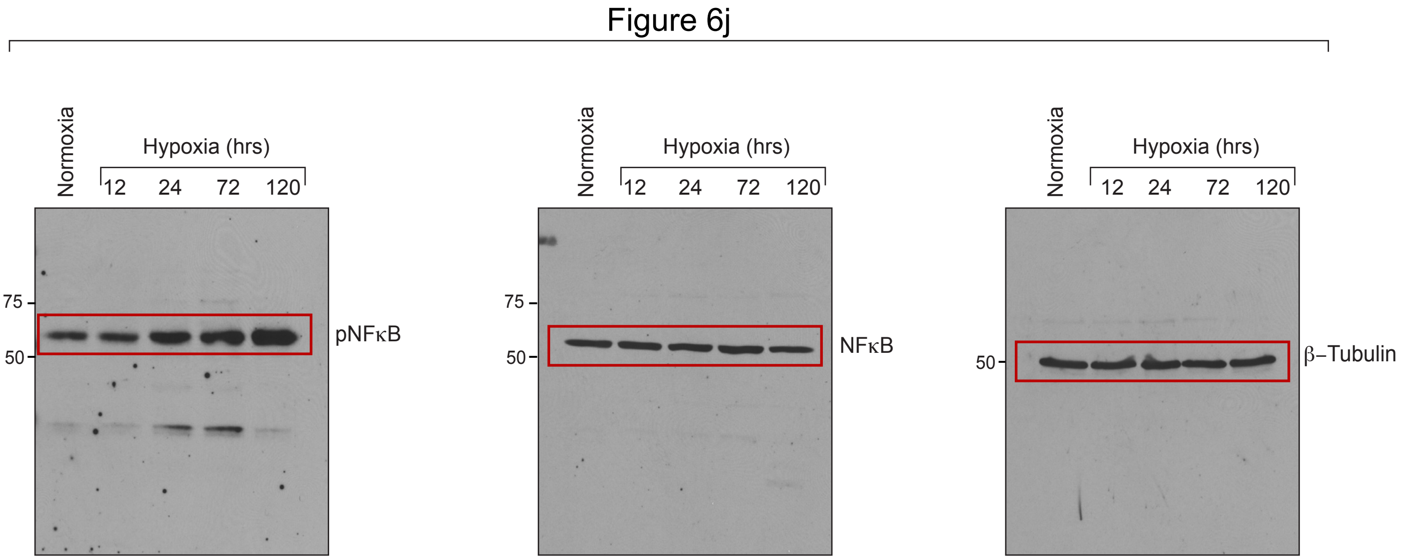

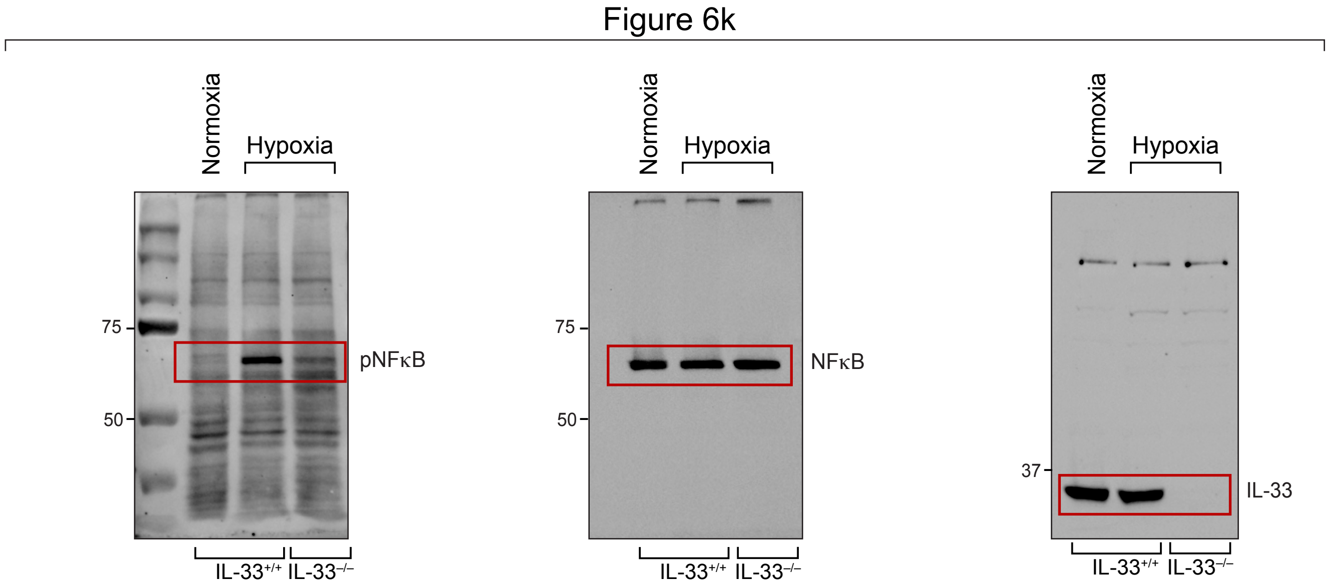

Supplementary Figure 7: Scans of immunoblots presented in Figure 7a & b.

Figure 7a

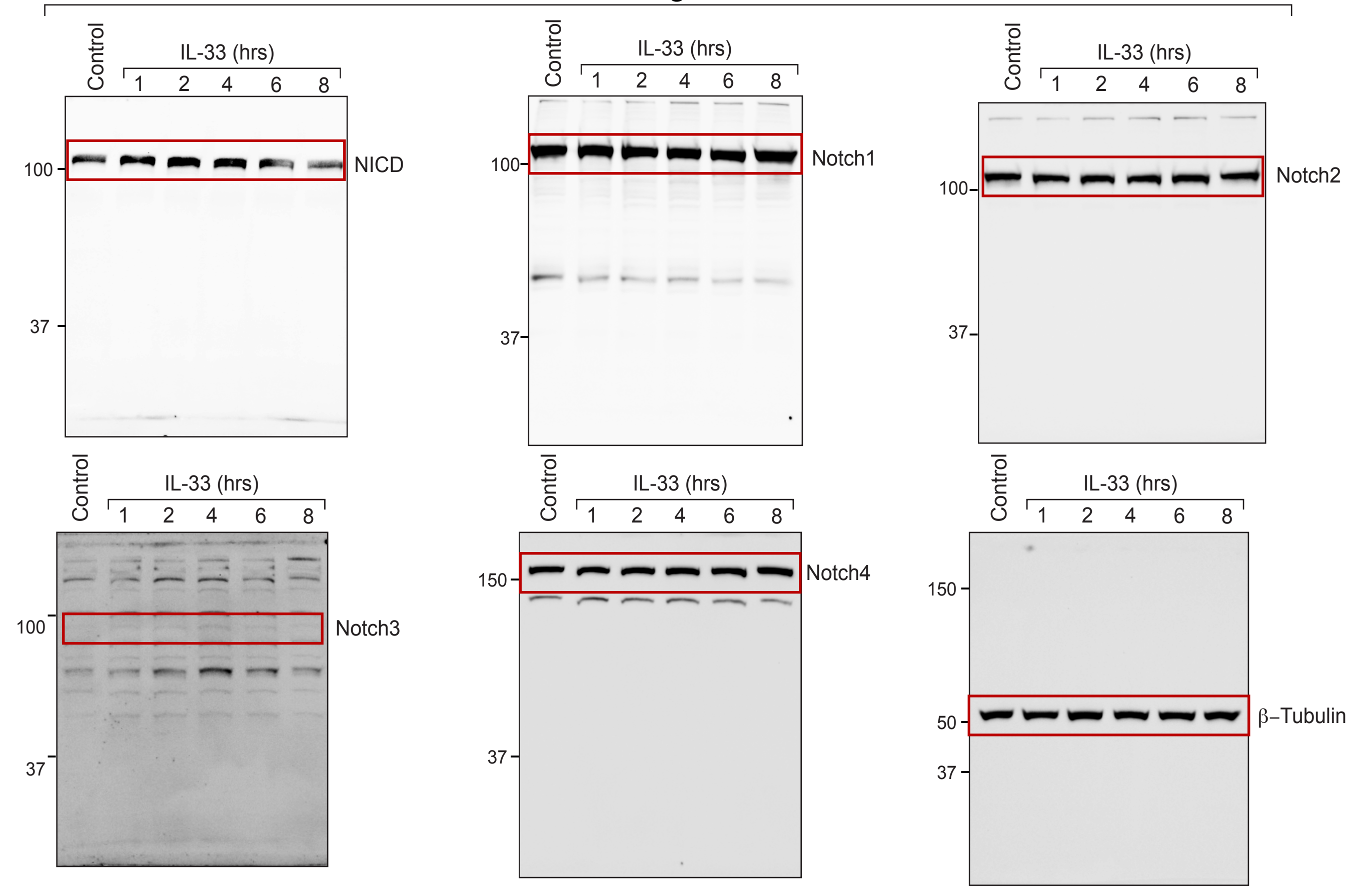

Figure 7b

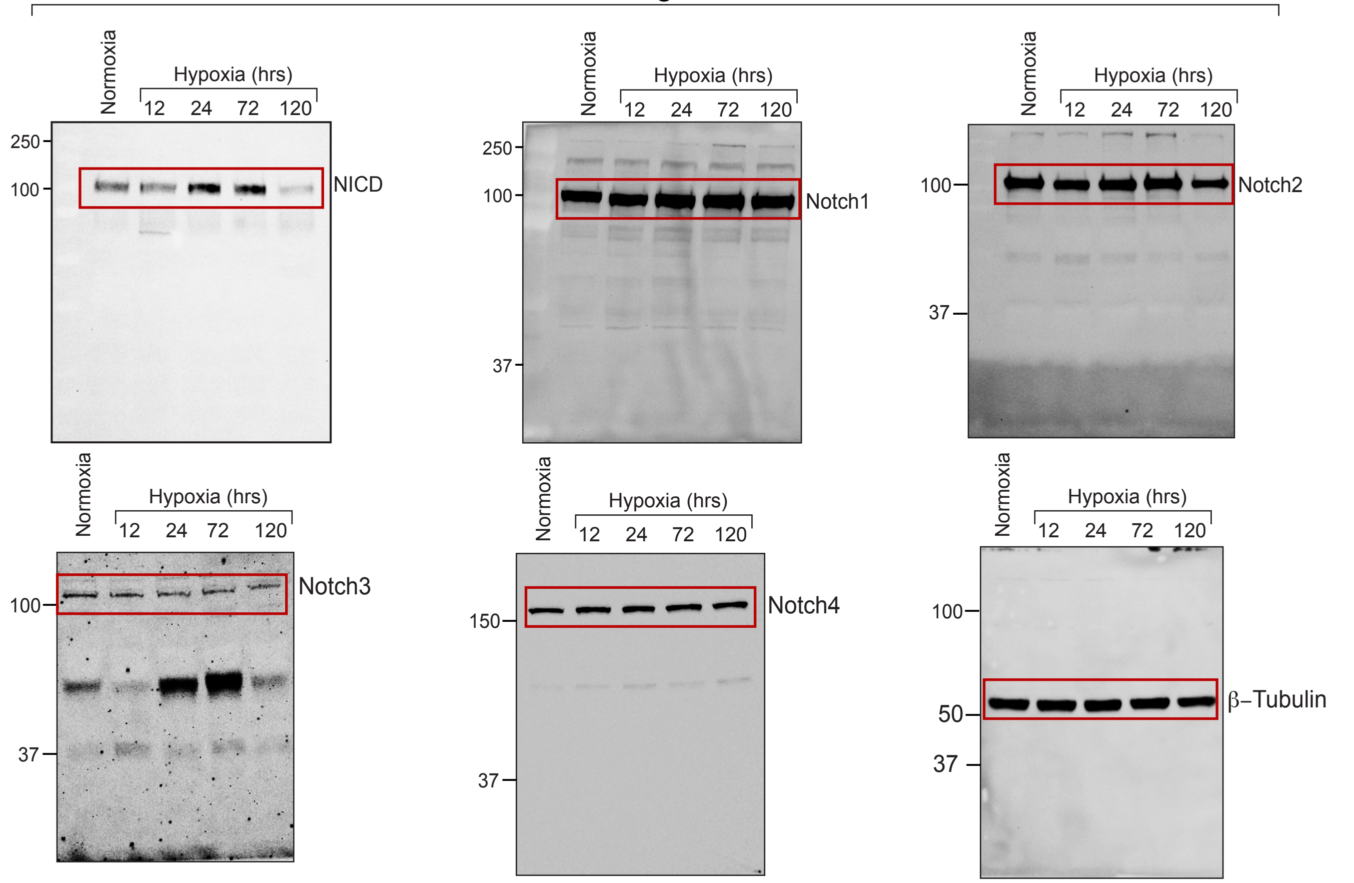

Supplementary Figure 8: Scans of immunoblots presented in Figure 7c & d.

Figure 7c

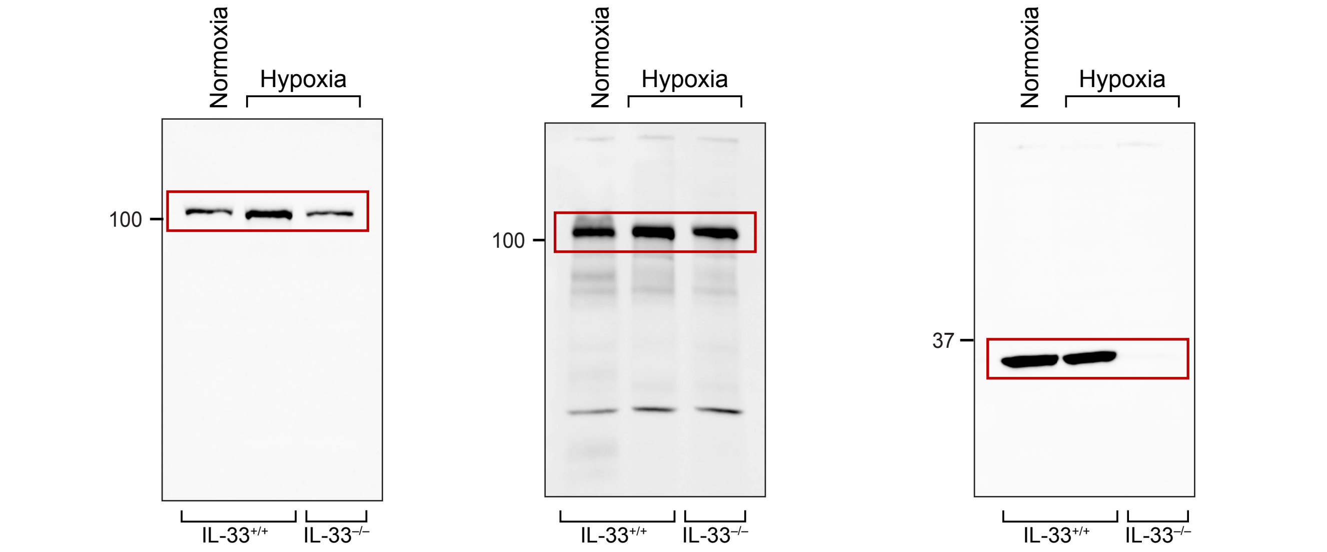

Figure 7d

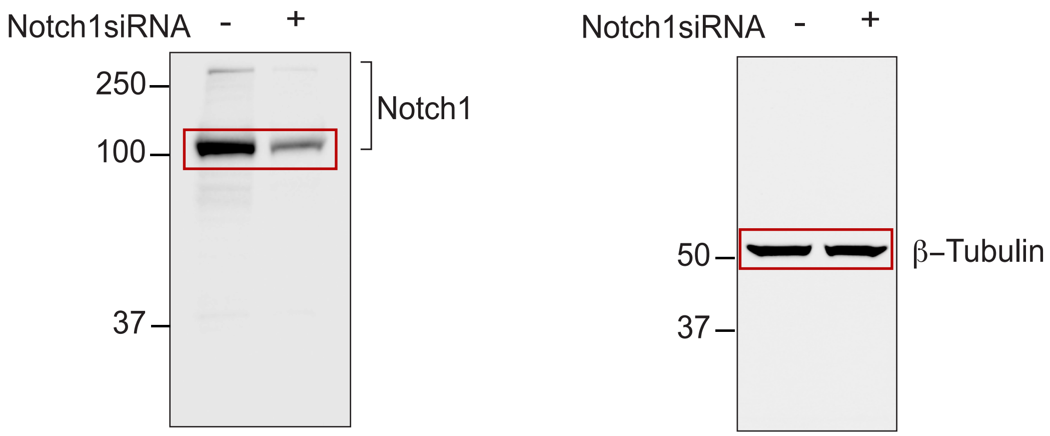

Supplementary Figure 9: Scans of immunoblots presented in Figure 8 b, c & d.

Figure 8a

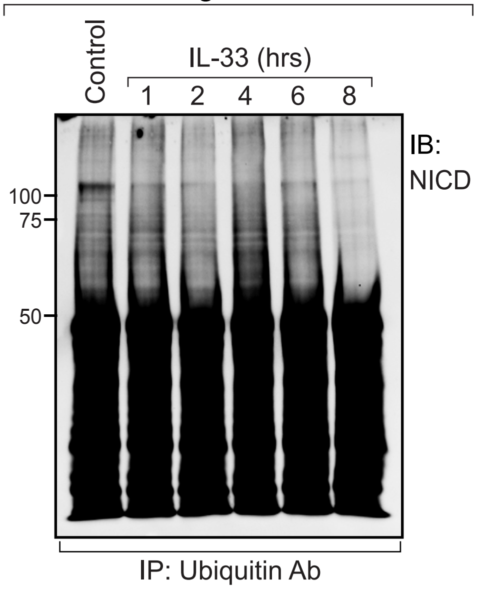

Figure 8b

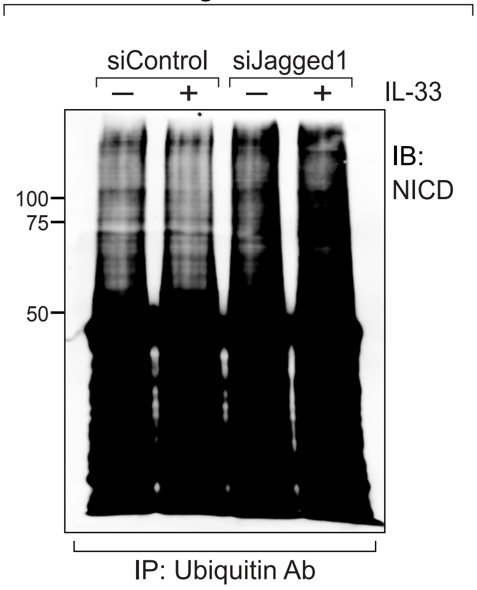

Figure 8c

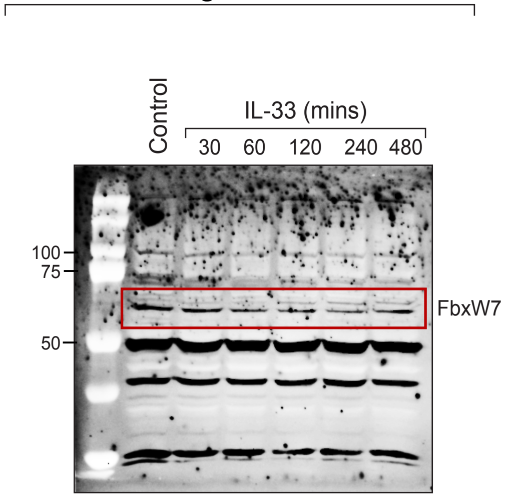

Figure 8c

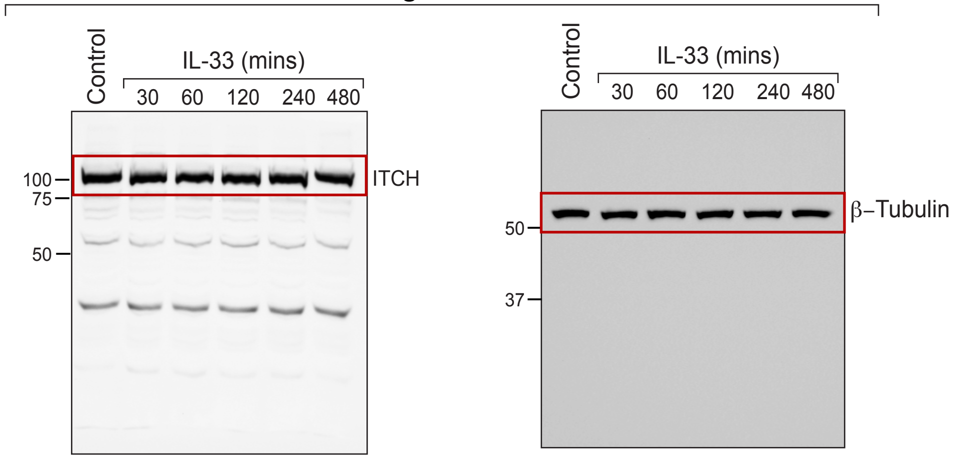

Figure 8d

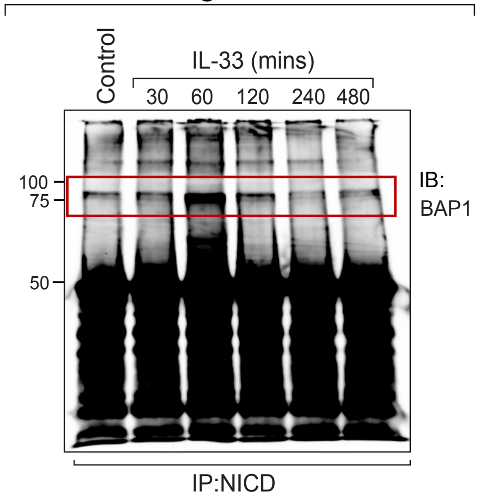

Figure 8d

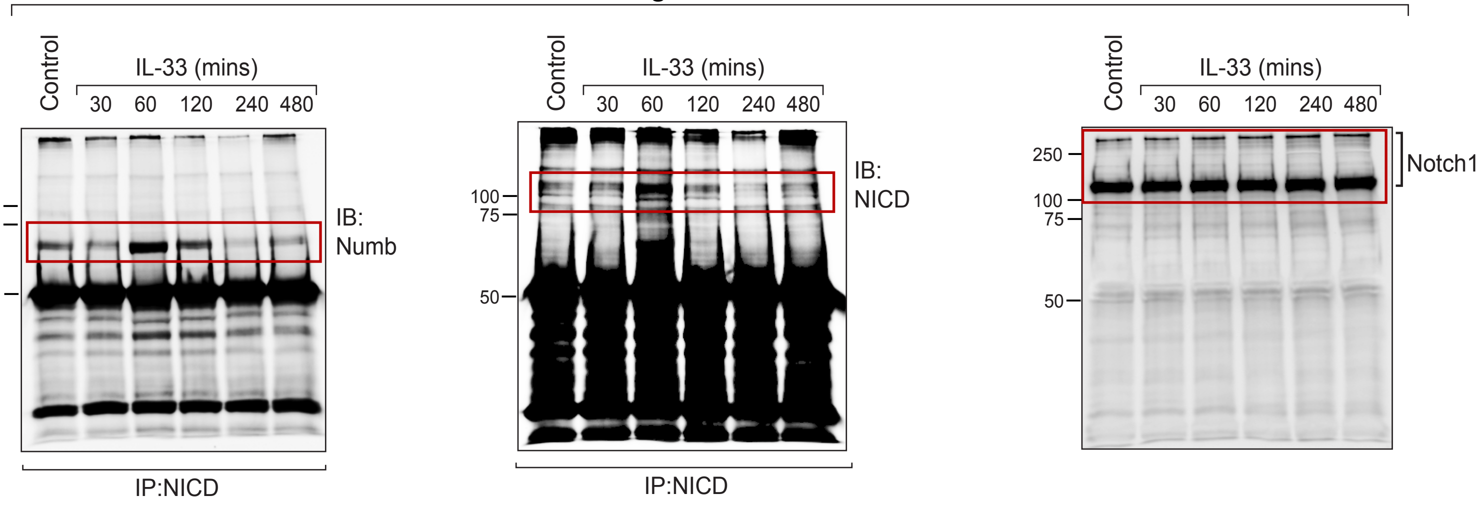

Supplementary Figure 10: Scans of immunoblots presented in Figure 8 e, & f and Suppl. Fig.2 a.

Figure 8e

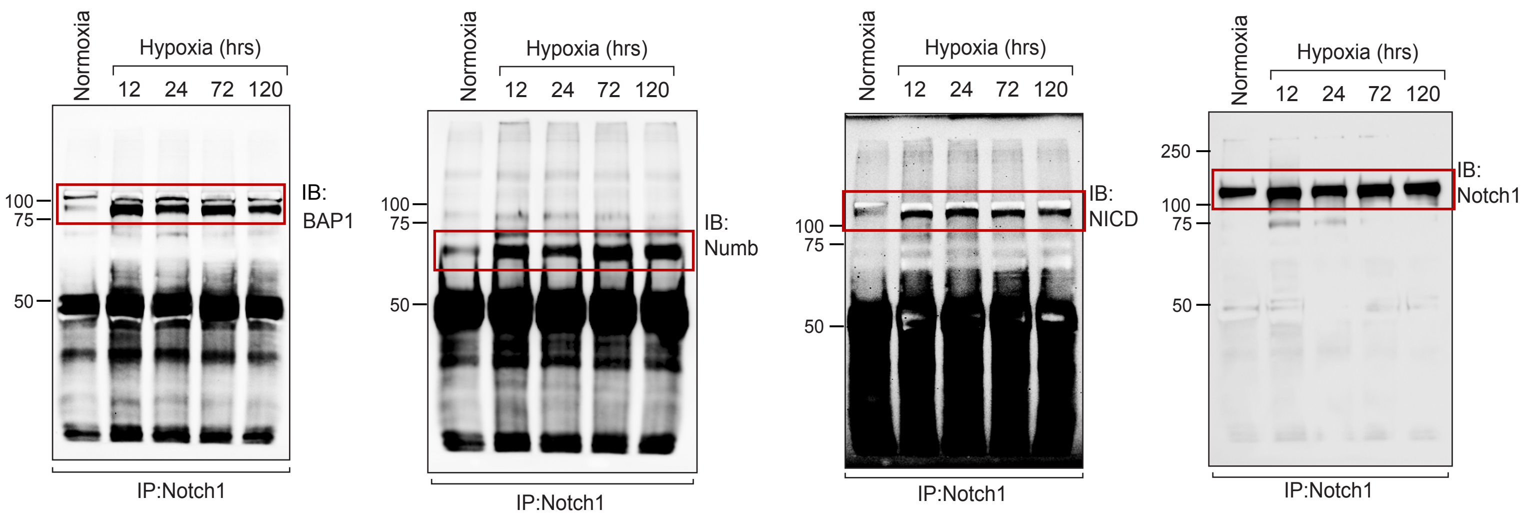

Figure 8f

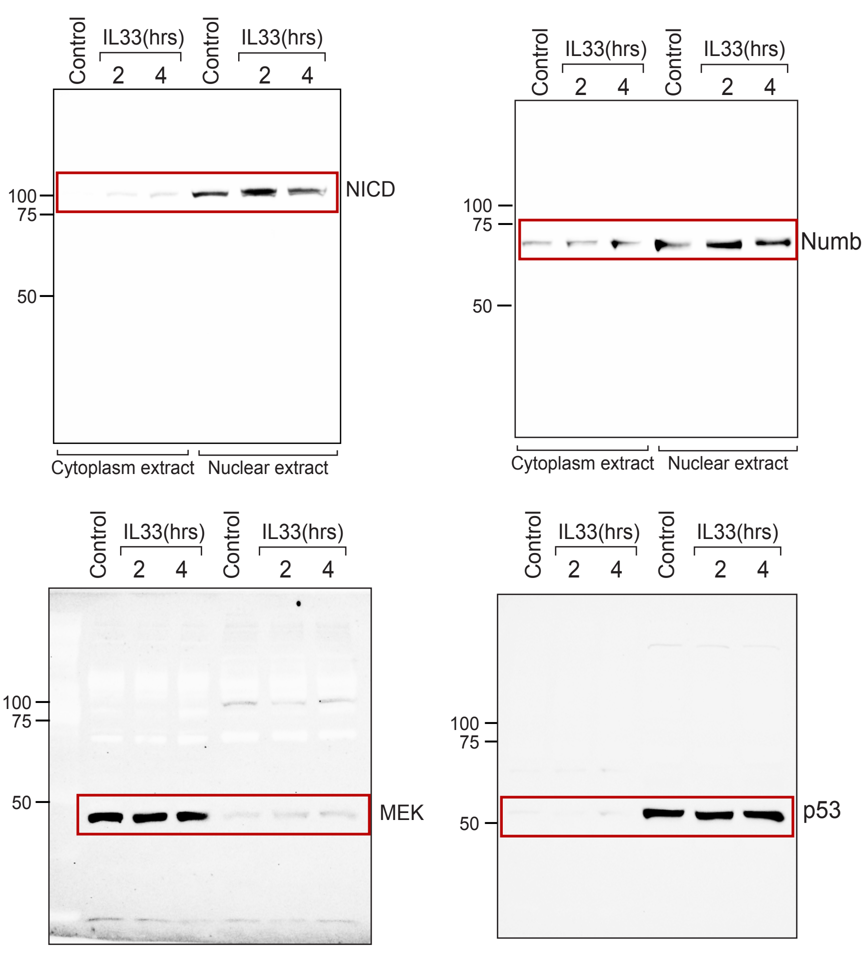

Suppl. Figure 2a

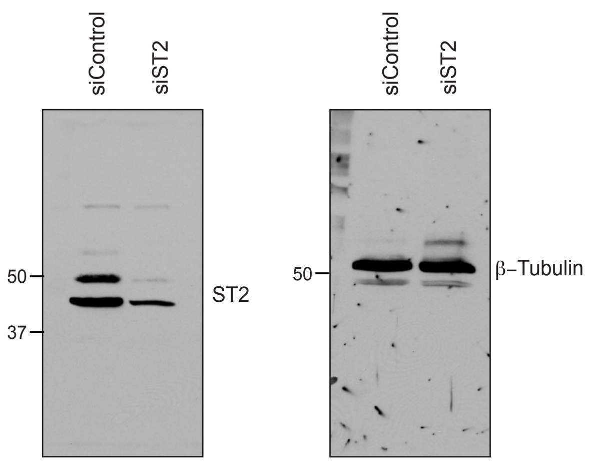

Supplement: Supplementary file 2 — Supplementary Information [file 42003_2022_3432_MOESM2_ESM.pdf]
