# Supplementary material for: IL-33 enhances Jagged1 mediated NOTCH1 intracellular domain (NICD) deubiquitination and pathological angiogenesis in proliferative retinopathy
Source: Commun Biol. 2022 May 19;5:479. doi: 10.1038/s42003-022-03432-7 (PMC9120174; doi:10.1038/s42003-022-03432-7)
Supplement: Supplementary file 3 — Description of Additional Supplementary Files [file 42003_2022_3432_MOESM3_ESM.pdf]

Description of Additional Supplementary Files

File name: Supplementary Data 1
Description: The statistical source data for all the graphs presented in figures.
